# Frontal recess anatomy study by endoscopic dissection in cadavers

**DOI:** 10.1016/S1808-8694(15)31067-3

**Published:** 2015-10-22

**Authors:** Marcus Miranda Lessa, Richards Louis Voegels, Bernardo Cunha Filho, Flavio Sakae, Ossamu Butugan, Gerald Wolf

**Affiliations:** 1 PhD in Sciences by the Otorhinolaryngology Discipline - FMUSP; Associate Researcher of Immunology and Otorhinolaryngology - HUPES - Federal University of Bahia; Fellow in Nasosinusal Endoscopic Surgery - Graz University - Austria. Mailing Address: Marcus Miranda Lessa - Rua João das Botas s/n Canela 40110-160 Salvador BA.; 2 Associate Professor of Otorhinolaryngology - Medical School - University of São Paulo.; 3 PhD student in Otorhinolaryngology - Medical School - University of São Paulo.; 4 PhD student in Otorhinolaryngology - Medical School - University of São Paulo.; 5 Associate Professor of Otorhinolaryngology - Medical School - University of São Paulo.; 6 Professor of Otorhinolaryngology - Graz University - Austria.; 7 Divisão de Clínica Otorrinolaringológica do Hospital das Clínicas da Faculdade de Medicina da Universidade de São Paulo.; 8 Serviço de Imunologia − 5^o^ andar Hospital Universitário Prof. Edgard Santos UFBA Fax: (0xx71) 245-7110

**Keywords:** endoscopy, frontal sinus, paranasal sinuses, frontal sinusitis

## Summary

I**ntroduction and Aims:** The frontal sinus ostium is frequently difficult to recognize because of anatomical structures that hide it. The objective of the present study was to identify and describe the frontal recess anatomy that impairs the endoscopic recognition of the frontal sinus ostium. **Study Design and Methods:** A prospective study was conducted by consecutive endoscopic dissections of 32 cadavers (59 sides), 10 (31.25%) females and 22 (68.75%) males. After resection of the lower portion of the uncinate process, with preservation of its upper insertion, we evaluated which anatomical structures needed to be removed for complete visualization of the frontal sinus ostium. **Results and Conclusions:** Visualization of the frontal sinus ostium after resection of the lower portion of the uncinate process was possible in only 11 (18.64%) nasal cavities. The uncinate process (terminal recess) was the main anatomical structure that impaired the recognition of the frontal sinus ostium, present in 45 (76.27%) nasal cavities, followed by the ethmoid bulla (16.95%) and agger nasi cells (6.78%).

## INTRODUCTION

The frontal sinus (FS) drainage path is made up of three different regions and is usually shaped like an hourglass. The upper portion of the hourglass is represented by the FS itself, while its narrowest portion corresponds to its ostium. The lower portion is formed by the frontal recess^1^, ^2^, ^3^.

The frontal recess (FR) is a space of variable development of the anterior pneumatized cells, which is usually called “duct” or “nasofrontal canal”1. FR walls and boundaries belong to adjacent structures, and its degree of patency is broadly determined by these structures^2^, ^3^, ^4^. Since this space extends to within the frontal sinus (FS), FS status is fully dependent on the FR conditions^1^.

Endoscopic functional surgery of the FS, which allows the selective treatment of the FR, presents results similar to the ones obtained by the external approaches, and recent papers report success rates between 82% and 90% for cases treated by this technique^5^,^6^. Nonetheless, FS and FR surgery still are considered the greatest challenge of nasosinusal endoscopic procedures. The obscure location of this area, anatomical variations and its close proximity with the eye and anterior cranial fossa may lead the surgeon to avoid its proper dissection, and it may also expose the patient to broader complications^7^.

A detailed knowledge of the lateral nasal wall and of the FR is essential for the success of FS functional endoscopic approach. Our major goal with the present study with endoscopic cadaver dissection was to identify and describe the frontal recess anatomical structures that impair the endoscopic identification of the frontal sinus ostium.

## MATERIALS AND METHODS

After approval by the Ethics Committee for the analysis of research projects (CAPPesq - Protocol # 414/02), we studied 32 cadavers (59 nasal cavities) at the São Paulo Morgue.

**Figure 1:**
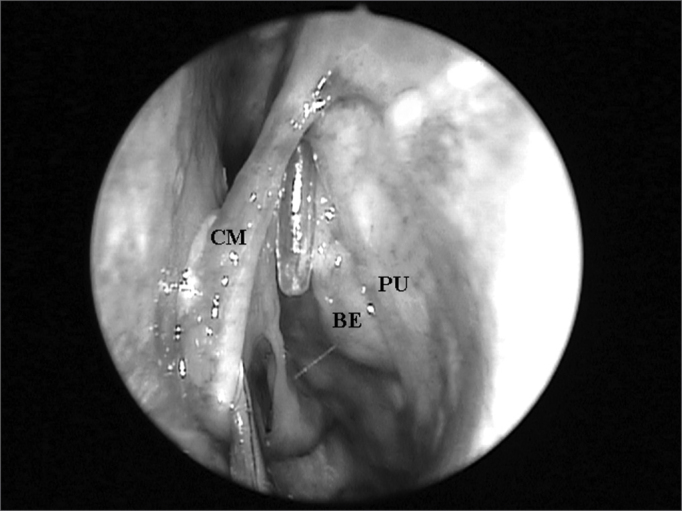
Left nasal cavity endoscopy showing the flexible plastic tube emerging from the middle meatus (CM = middle turbinate; PU = unciform process; BE = ethmoidal bulla).

For this study we used the following exclusion criteria: age below 20 years; bilateral frontal sinus agenesis; prior history of nasal trauma; history of nasosinusal surgery; nasosinusal disorders that distorted local anatomy (e.g. nasal polyps and tumors). FS presence was assessed by means of frontal bone osteotomy in its lowest possible point, that is, immediately above and medial to the ipsilateral orbit border. In the presence of a FS, we catheterized its ostium under direct visualization by means of a flexible plastic tube, all the way down to the middle meatus (Figure 1).

**Figure 2:**
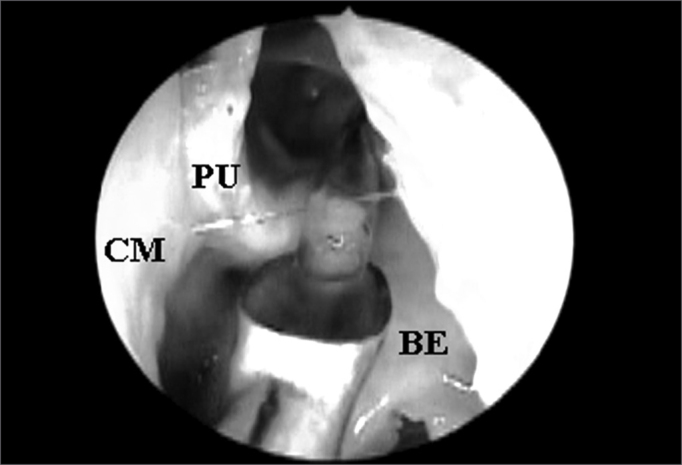
Left nasal cavity endoscopy with 4mm 45^o^ rigid endoscope showing the direct visualization of the frontal nasal ostium catheterized by the flexible plastic tube after the removal of the unciform process inferior portion (CM = middle turbinate; PU = unciform process; BE = ethmoidal bulla).

After endoscopic incision and exeresis of the unciform process (UP) by means of a straight cutting forceps, preserving its upper portion, we assessed FS ostium endonasal visualization, which was catheterized by the flexible plastic tube, without the need to remove any other anatomical structure (Figure 2). For this end, we used a 4mm 45^o^ rigid endoscope, positioned in the middle meatus and guided upwards to the FR. When such direct visualization of the FS ostium was not possible, we identified the anatomical structures that needed to be removed so as to allow its complete visualization.

Data was divided in groups according to gender and race. In the latter, we grouped the Caucasians and compared to Brown and Black individuals, together. Gender and race data analysis was carried out by using nominal data. We used continuous data in order to quantify the number of structures that prevented the frontal sinus ostium visualization. Variable distribution analysis was carried out by using the Komogorof-Smirnov normality test. In order to compare the frontal sinus ostium endoscopic visualization in relation to gender, we used the Fisher exact test; and for race, we used the chi-squared. In order to investigate the number of structures precluding frontal sinus ostium endoscopic visualization with gender and race, we used the Student t test. We considered 95% as significance index (p<0.05).

## RESULTS

**Graph 1:**
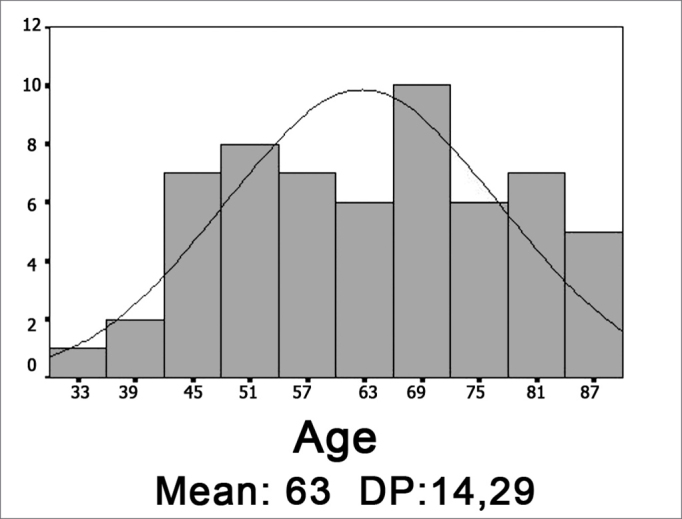
Age histogram (DP = standard deviation).

**Graph 2:**
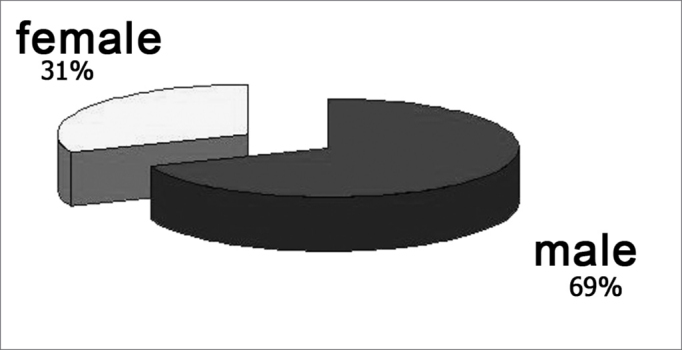
Cadaver classification as to gender.

**Graph 3:**
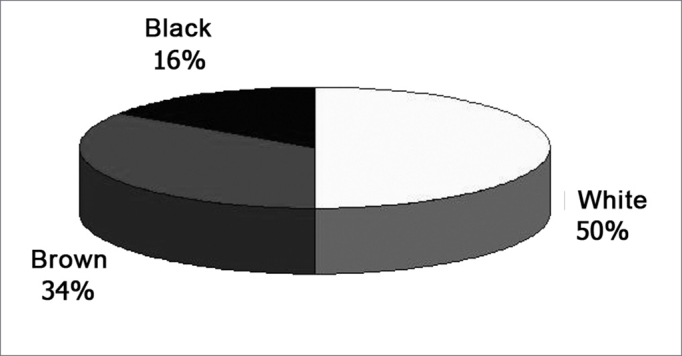
Cadaver classification as to race.

The cadavers' ages ranged between 30 and 88 years, and both the average and standard deviation are shown in Graph 1. As to gender, most were males (graph 2). Half the cadavers were Caucasians, and the detailed race distribution is shown in Graph 3.

Of the 32 cadavers dissected, five (15.63%) had unilateral FS agenesis. Therefore, FS ostium endonasal visualization assessment was carried out in 59 nasal cavities.

Direct FS ostium visualization with the 4mm 45^o^ rigid endoscope positioned in the middle meatus and guided upwards to the FR, immediately after removing the lower portion of the UP was possible in 11 (18.64%)

nasal cavities. On the remaining 48 (81.36%) nasal cavities, the FS ostium shown by the flexible plastic tube was not clearly seen, and the anatomical structures that impaired this visualization are described below. When the frontal sinus ostium endonasal visualization, after the removal of the unciform process inferior portion, was compared gender wise, we observed that it was more frequent in males, although there was no statistical difference (p=0.079). Of the 11 nasal cavities in which it was possible to visualize the FS ostium, 10 were from males. We did not find association between race and the capacity to directly see the FS ostium (p=0.88).

The UP inserted in the lamina papyracea (terminal recess), seen in 45 (76.27%) nasal cavities, was the main anatomical structure that impaired FS ostium endonasal visualization among the 59 nasal cavities evaluated (Figures 3A and 3B), followed by the ethmoidal bulla (Figures 4A and 4B) which was present in 10 (16.95%) nasal cavities and the agger nasi cells (Figures 5A and 5B) in 4 (6.78%) nasal cavities.

**Figure 3A:**
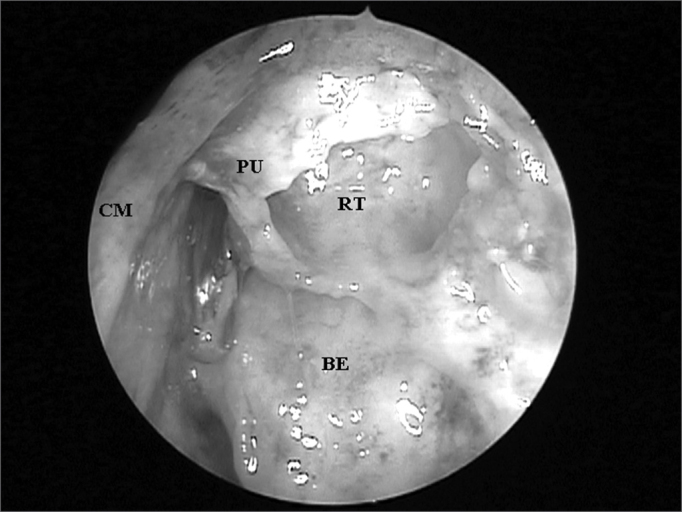
Left nasal cavity endoscopy with 4mm 45^o^ rigid endoscope showing the terminal recess that impairs the direct frontal sinus ostium visualization;

**Figure 3B:**
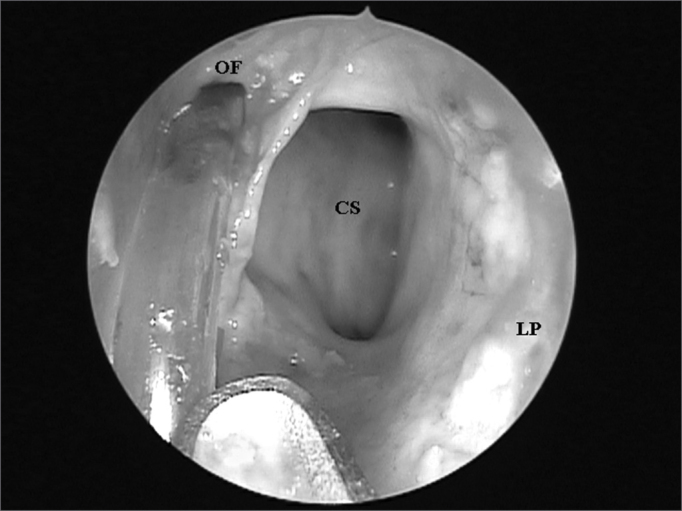
Frontal sinus ostium catheterized by the flexible plastic tube and located medially to a supraorbitary cell after terminal process exeresis ((CM = middle turbinate; PU = unciform process; BE = ethmoidal bulla; OF = frontal sinus ostium; CS = supraorbitary cell; RT = terminal recess; LP = lamina papyracea).

**Figure 4A:**
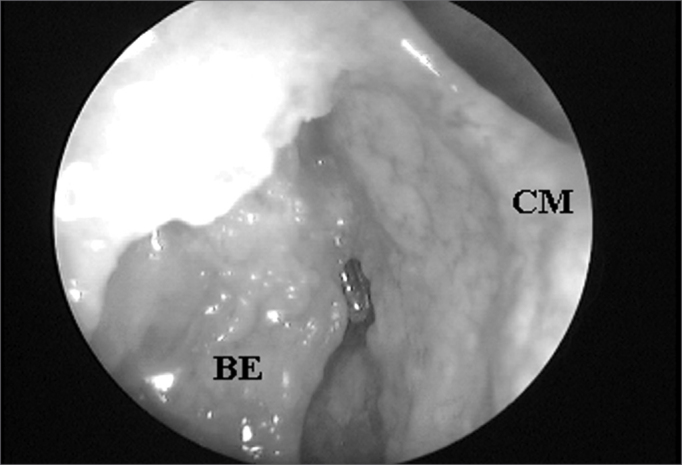
Right side nasal cavity endoscopy with 4mm 45^o^ rigid endoscope showing the ethmoidal bulla impairing the endonasal visualization of the frontal sinus ostium;

**Figure 4B:**
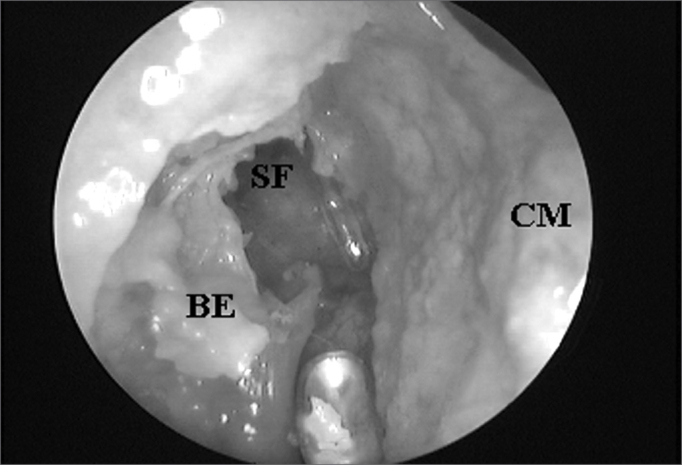
Ethmoidal bulla superior portion exeresis and consequent frontal sinus ostium visualization when catheterized by the flexible plastic tube (CM = middle turbinate; BE = ethmoidal bulla; SF = frontal sinus).

**Figure 5A:**
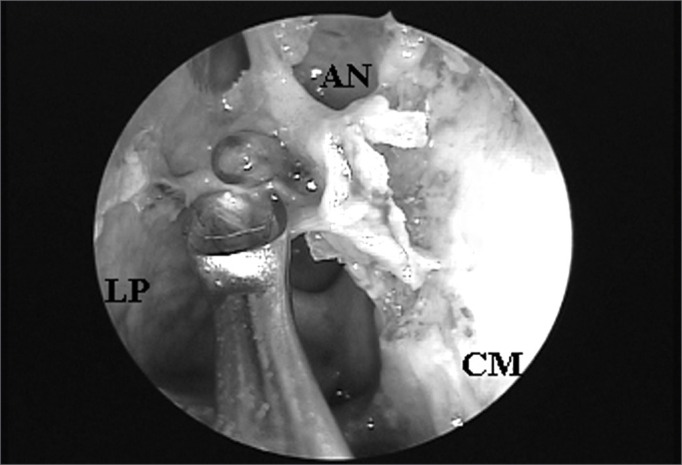
Right nasal cavity endoscopy with a 4mm 45^o^ rigid endoscope showing an agger nasi cell impairing endonasal frontal sinus ostium visualization.

**Figure 5B:**
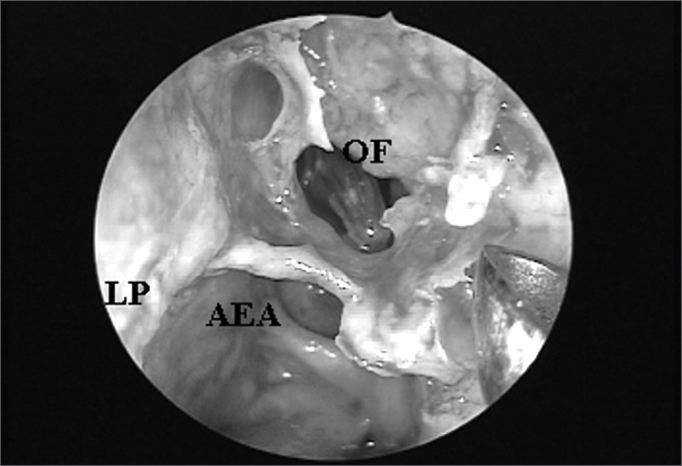
Agger nasi cell exeresis and consequent frontal sinus ostium catheterized by the flexible plastic tube visualization (CM = middle turbinate; LP = lamina papyracea; AN = agger nasi; OF = frontal sinus ostium; AEA= anterior ethmoidal artery).

In 11 (18.64%) of the 59 nasal cavities, there was more than one anatomical structure covering the FS ostium, and the unciform process together with the ethmoid bulla were associated in nine (15.25%) nasal fossae, being the most frequent obstacle. The other association found was between the unciform process and the agger nasi, shown in two (3.39%) nasal fossae.

Comparing Caucasians with Brown and Black individuals as far as the number of structures preventing FS ostium endonasal visualization is concerned, we did not observe statistically significant difference (p=0.68), fact that was also observed when the groups were compared as to gender (p=0.31).

## DISCUSSION

According to Caliot et al.8 (1990), the FS ostium is almost always located deep and is difficult to see. This statement is in agreement with the present study, in which direct visualization of the FS ostium with the 45^o^ endoscope, immediately after removing the UP inferior portion was only possible in 11 (18.64%) of the 59 nasal cavities evaluated. In the 48 (81.36%) remaining nasal fossae, the FS ostium was covered by some anatomical structure. It is probably because of this large difficulty in visualizing the FS ostium that Stamberger4 (1999) states that the endonasal access to the FS ostium requires deep anatomical knowledge and large surgical skill and accuracy.

The UP was the major anatomical structure that prevented endonasal visualization of the FS ostium among the 59 nasal cavities studied. In 45 (76.27%) nasal cavities, the UP superior insertion was present covering up the FS ostium, alone or in association with other anatomical structures. As we analyze these results, we may infer that similar to the maxillary sinus natural ostium, which may be exposed after removing the lower portion of the UP, the FS ostium may, most of the times, be exposed after removing the UP upper portion. Thus, we agree with Friedman et al.^7^ (2000) when they state that identifying UP anatomical variations and its accurate removal in the upper portion allow surgical access to the FS by the identification of its natural ostium, what increases the chance of it remaining patent. Also fundamental is Lee et al's.^9^ (1997) observation that the surgeon must first inspect the FR anatomy before removing the UP (it is normally necessary to push the middle turbinate), and also after its removal, but before completing the ethmoidectomy. The variations in relation to FS drainage must be indicated and the adjacent structures should be analyzed.

The meaning of UP insertion in the lamina papyracea had already been observed by Kasper10 since 1936, when he wrote that the knowledge of this frequent blind cul-de-sac in the ethmoidal infundibulum is important for the surgeon who will operate on noses. Our results further reinforce this statement by Kasper^10^, because besides this terminal recess being frequently found in the population, its identification became fundamental for a correct dissection and access to the FS ostium, and such dissection is carried out medially to the UP.

The extensive pneumatization in the EB, posterior to the FR and the agger nasi, anterior to this recess, predispose patients to an appearance similar to a duct in saggital cross-sections that is why it is incorrectly named “nasofrontal duct” by some authors. Many frontal recesses seem to drain superior and medially to the ethmoidal infundibulum, some drain directly to this infundibulum and just a few drain to the retrobullar recess^2^. The retrobullar recess is the space located between the EB and the middle turbinate basal lamella, also called lateral sinus. Since 1936, KASPER10 had reported FS drainage to the retrobullar recess in 2% of the cases. He called the attention to the fact that this condition, although rare, causes a difficult endonasal access to the FS, especially because of a steep angle stemming from the BE projection. Similarly to KASPER10 (1936), Van Alyea11 (1946) and Kim12 (2001) observed that in only 1% of the cases the FS drains directly to the retrobullar recess.

The EB was the second anatomical structure that most impaired the endonasal visualization of the FS ostium, fact that occurred in 10 (16.95%) among the 59 nasal fossae evaluated. In 4 (6.78%) cases, the FS ostium was draining to the retrobullar recess, and in 6 (10.17%) cases, the ethmoidal bulla hyperpneumatization made impossible the endonasal identification of the frontal sinus ostium.

Since most of the times the FR posterior wall is formed by the EB basal lamella, we agree with Loury^13^ (1993) when he states that the intact EB besides protecting the anterior ethmoidal artery, is an excellent anatomical reference point for recess and FS the posterior margin, and whenever possible it should be preserved during the FR endoscopic surgical approach. We would just like to highlight the fact that the EB may eventually represent the FR anterior wall or its anterior pneumatization that may cover the frontal ostium, thus, if one can not identify the FS ostium after proper and careful dissection of the UP in its antero-superior portion, EB removal must necessarily be the next step in surgery.

The agger nasi, the most anterior ethmoidal pneumatized cell is present in over 98% of the patients. This cell may pneumatize posteriorly, towards the FR, thus causing problems related to FS obstruction that varies from an asymptomatic mucocele to headaches and other sinus diseases. Agger nasi cell location and level of pneumatization vary enormously14. Sometimes it is difficult to differentiate an agger nasi cell from a high terminal recess, because both appear antero-superiorly as a cul-de-sac15. Although the agger nasi is very constant and present in the human being nasosinusal anatomy, its role in impairing the FS ostium visualization was less pronounced in our study (6.78% of the cases).

## CONCLUSION

The unciform process inserted in the lamina papyracea (terminal recess) represented the major anatomical structure that made it difficult to recognize the frontal sinus ostium, followed by the ethmoidal bulla and the agger nasi cell.
